# Systemic inflammation is negatively associated with early post discharge growth following acute illness among severely malnourished children - a pilot study

**DOI:** 10.12688/wellcomeopenres.16330.2

**Published:** 2021-03-16

**Authors:** James M. Njunge, Gerard Bryan Gonzales, Moses M. Ngari, Johnstone Thitiri, Robert H.J. Bandsma, James A. Berkley

**Affiliations:** 1 The Childhood Acute Illness & Nutrition (CHAIN) Network, Nairobi, Kenya; 2 KEMRI/Wellcome Trust Research Programme, Kilifi, Kenya; 3 Department of Internal Medicine and Pediatrics, Faculty of Medicine and Health Sciences, Ghent University, Ghent, Belgium; 4 Centre for Global Child Health, The Hospital for Sick Children, Toronto, Ontario, Canada; 5 Centre for Tropical Medicine & Global Health, Nuffield Department of Medicine, University of Oxford, Oxford, UK

**Keywords:** severe malnutrition, child growth, weight, mid-upper arm circumference, anthropometric deficit, inflammation, cytokines, proteome

## Abstract

**Background: **Rapid growth should occur among children with severe malnutrition (SM) with medical and nutritional management. Systemic inflammation (SI) is associated with death among children with SM and is negatively associated with linear growth. However, the relationship between SI and weight gain during therapeutic feeding following acute illness is unknown. We hypothesised that growth post-hospital discharge is associated with SI among children with SM.

**Methods:** We conducted secondary analysis of data from HIV-uninfected children with SM (n=98) who survived and were not readmitted to hospital during one year of follow-up. We examined the relationship between changes in absolute deficits in weight and mid-upper-arm circumference (MUAC) from enrolment at stabilisation to 60 days and one year later, and untargeted plasma proteome, targeted cytokines/chemokines, leptin, and soluble CD14 using multivariate regularized linear regression.

**Results: **The mean change in absolute deficit in weight and MUAC was -0.50kg (standard deviation; SD±0.69) and -1.20cm (SD±0.89), respectively, from enrolment to 60 days later. During the same period, mean weight and MUAC gain was 3.3g/kg/day (SD±2.4) and 0.22mm/day (SD±0.2), respectively. Enrolment interleukins; IL17-alpha and IL-2, and serum amyloid P were negatively associated with weight and MUAC gain during 60 days. Lipopolysaccharide binding protein and complement component 2 were negatively associated with weight gain only. Leptin was positively associated with weight gain. Soluble CD14, beta-2 microglobulin, and macrophage inflammatory protein 1 beta were negatively associated with MUAC gain only. Glutathione peroxidase 3 was positively associated with weight and MUAC gain during one year.

**Conclusions:** Early post-hospital discharge weight and MUAC gain were rapid and comparable to children with uncomplicated SM treated in the community. Higher concentrations of SI markers were associated with less weight and MUAC gain, suggesting inflammation negatively impacts recovery from wasting. This finding warrants further research on reducing inflammation on growth among children with SM.

## Introduction

In 2018, approximately 50.5 million children under five years old globally were wasted, of which 16 million were severely wasted ^1, 2^. Wasting is associated with elevated mortality, mainly due to susceptibility to infectious diseases ^3– 5^. Current guidelines recommend that children with severe wasting or oedematous malnutrition who are acutely ill (complicated severe malnutrition; CSM) are initially medically treated and nutritionally stabilised as inpatients. Once stabilised, they are treated with high protein and energy feeds in the form of ready-to-use therapeutic foods (RUTF) to achieve catch-up weight gain as outpatients ^6, 7^.

Severely malnourished children admitted to hospital with acute illness may suffer relapse, readmission, or death after discharge from hospital ^8– 13^ and are at risk of impaired neurocognitive development ^14– 16^. Children may return to household settings of poverty, social disadvantage, environmental contamination, and inadequate access to healthcare ^17– 25^. Enhanced prevention of recurrent illnesses over longer periods following hospitalisation and improved dietary quality have been suggested as opportunities to improve growth ^10, 11^.

RUTF was designed to fulfil 100% of the nutritional needs of children recovering from SM and may theoretically enable weight gain of up to 20 g/kg/day ^26^. Weight gain velocity is usually high at the start of the therapeutic feeding, then decreases and later plateaus ^27– 34^. Weight gain may be affected by comorbidities such as HIV or other chronic infections but may also be related to intestinal or systemic inflammation (SI), leading to reduced appetite, nutrient malabsorption, and metabolic changes ^25, 35, 36^.

SI is demonstrable at the time of hospital discharge in children with SM ^37^. However, it is not known how long inflammation persists or what its effects are on weight gain. In high income settings, survivors of sepsis have elevated markers of SI for up to one year and retain an increased mortality risk ^38– 42^. After sepsis, systemic levels of C-reactive protein (CRP) and soluble programmed death ligand 1 (a marker of immunosuppression) are elevated for up to one year in patients while interleukin 6 (IL6) and IL10 persists for several months in human and experimental models ^38– 40^. SI, characterised by elevated cytokines including IL1β and IL6, is known to suppress linear growth indirectly through the growth hormone/insulin growth factor 1 (GH/IGF1) axis, and directly through effects on long bone growth plate chondrocytes ^25, 43^. In Nepal, birth size was inversely associated with low-grade, chronic inflammation during pregnancy as indicated by serum Alpha 1 acid Glycoprotein ^44, 45^. Further, head size at birth among Nepalese ^46^ and attained z scores for height (HAZ) and weight-for-age (WAZ) at 6–8 years of age ^47^ were associated with a wide array of plasma proteins, including S100 calprotectin subunits, assessed at 6–8 years. In community settings, SI is elevated in 17–34% children in LMIC ^48^ (CRP >5mg/L or α1-antichymotrypsin >0.6g/L) and is associated with reduced linear growth ^49– 53^. Besides linear growth, SI may affect gain in adipose and muscle through promoting a persistent catabolic state and dysregulation of the usual hormonal and metabolic processes of these tissues ^54– 56^.

Both nutrient scarcity and acute illness are associated with a catabolic state ^55^ with negative effects on the body’s storage organs, mainly adipose and muscle. During refeeding of children with SM, significant systemic metabolic shifts are observed that relate to the muscle, liver, and the adipose tissue among others ^57, 58^. We therefore hypothesised that among children with SM treated in hospital for an acute illness, weight gain is associated with SI. The objective of this study was to investigate the relationship between plasma proteomic and cytokine profiles and weight gain among HIV negative children with SM in the first 60 days of post-hospital discharge following medical stabilisation.

## Methods

### Ethics approval and consent to participate

The trial was approved by the Kenya National Ethical Review Committee (SSC 1562) and the Oxford Tropical Research Ethics Committee (OXTREC reference 18–09). Secondary analyses were approved by the Scientific and Ethical Review Unit (SERU 2782). The trial was registered at clinicaltrials.gov ( NCT00934492, 8 ^th^ July 2009). Informed consent for data and sample collection, storage, and future research had been obtained from mothers or guardians of study participants during recruitment to the trial.

### Study design and patient recruitment

This was a secondary analysis of data from a nested case control study ^37^ within a clinical trial ( NCT00934492) that tested the efficacy of daily co-trimoxazole prophylaxis in reducing post-discharge mortality among HIV-uninfected children aged 2–59 months hospitalised with CSM in two urban (Mombasa and Nairobi) and two rural (Kilifi and Malindi) hospitals in Kenya ^8^. Children were included in the trial if they had mid-upper-arm circumference (MUAC) <11.5cm if aged ≥6 months and <11.0cm if aged 2–5 months or had oedematous malnutrition; and had a negative HIV rapid-antibody test; and had completed the stabilisation phase of treatment as defined in WHO guidelines. Children were enrolled just prior to discharge from hospital. Samples were collected from study participants prior to initiation of the investigational product: co-trimoxazole or placebo at discharge and constitute enrolment samples. Discharge was according to WHO guidelines, based on clinical recovery rather than achieving an anthropometric threshold. At hospital discharge, nutritional counselling was given to caregivers, along with RUTF dosed as per WHO and Kenyan guidelines, and families were actively referred to community-based management of acute malnutrition (CMAM) centres located either at the hospital or in community facilities to continue therapeutic feeding. Children were actively followed up for 12 months, monthly in the first six months, and at months eight, 10 and 12. Study participants were traced at home if they defaulted and loss to follow-up was minimal (≤5%). The trial intervention had no overall effect on reducing mortality or hospital readmission.

Participants selected for this study had served as controls in a previous case control study ^37^. Briefly, the case to control ratio in the case control study was 1:1 and there were 121 cases (deaths) that were analysed that had sufficient samples from among 147 deaths that had occurred within the first 60 days of enrolment into the trial. Control children (n=120) had been randomly selected without replacement amongst 1119 children who survived and were not readmitted to hospital during 12 months of trial follow up using the ‘ *sample*’ command in STATA (version 15.1, TX, USA). For this study, 12 children who were oedematous at enrolment and another 10 children that lacked anthropometry data at month 2 to month 6 were excluded from the analysis. We therefore analysed data for 98 children in which plasma proteomic and cytokine measurements had been done on enrolment samples. 

### Data sources and measurements

During enrolment and at follow-up, child and caregiver demographic characteristics, immunisation status, clinical examination, admission diagnoses, chronic conditions, and anthropometry (weight, height or length, MUAC) were collected ^8^. Weight was measured with the use of an electronic scale (Seca 825), length or height with the use of an infantometer (Seca 416) or stadiometer (Seca 215), and MUAC with the use of insertion tape (TALC) ^8^. The WHO (2006) growth references were used to calculate Z scores.

### Proteomics and cytokines measurement in plasma

Untargeted plasma proteomics were measured by liquid chromatography tandem mass spectrometry and targeted cytokines, chemokines, leptin and soluble CD14 (sCD14) by Luminex and ELISA as previously described ^37^.

### Bioinformatics and statistical analysis

The primary and secondary outcomes were the change in absolute deficits in weight (DWAD) and MUAC (DMAD), respectively, from enrolment to 60 days. We further analysed the change in absolute weight, MUAC and height deficits from enrolment to 1 year. Absolute deficit was defined as the median value for age according to WHO growth charts minus the child’s measured value. It was calculated as the difference between the measured weight, MUAC, or height and the median age- and sex-specific value obtained from the WHO 2006 growth standards ^59– 61^. Absolute deficit was used rather than Z scores for weight-for-age (WAZ) or weight-for-height (WHZ) because changes in standard deviation across age or length makes them less appropriate for measuring changes over time among children of different ages ^60^. Exposure variables were the plasma proteome, leptin, sCD14 and a panel of targeted cytokines that are markers of inflammation and immune activation. Regression models were adjusted for age, sex, randomisation and site, whilst regression to the mean was addressed by including enrolment anthropometric values in the regression models. We hypothesised that proteins measured at baseline would have their strongest effect on early growth (within 60 days) than at later time points. We conducted the analysis in the R statistical software version 3.6.2 ^62^ and performed a multivariate regularized linear regression analysis using an elastic net (EN) model implemented using the “glmnet” package. This package fits a generalized linear model via penalized maximum likelihood. EN is a penalized regression approach and integrates two regularized approaches, ridge regression and LASSO (Least Absolute Shrinkage and Selection Operator), wherein the contribution of each of these models to the final EN model is controlled by the α parameter ^63, 64^. The EN penalty is controlled by α and bridges the gap between LASSO (α=1, the default) and ridge (α=0). The tuning parameter lambda (λ) that controls the overall strength of the penalty was determined using five-fold cross validation. The strong penalization imposed by LASSO draws non-predictive coefficients to zero, thereby eliminating proteins from the models, whereas ridge regression addresses potential multi-collinearity problems in high-dimensional data ^63, 64^. Variables such as age, sex, randomisation arm and site were treated as prior confounders and were not subjected to penalization by imposing a penalty factor of 0. All other variables had a penalty factor of 1 and were subjected to penalization. We used the ‘caret’ package in R to automatically select the best tuning parameters alpha and lambda by testing a range of possible alpha and lambda values. The best alpha and lambda values are those values that minimize the cross-validation error. EN model generation was performed separately for each growth outcome: change in Weight Absolute Deficit (DWAD) (primary outcome) and change in MUAC Absolute Deficit (DMAD) (secondary outcome), with protein profiles, cytokines, and enrolment anthropometric variables as predictors. As a further analysis, EN models were generated for DWAD, DMAD and Height Absolute Deficit (DHAD) for changes during one year. The subset of variables assigned non-zero coefficients were considered optimal and were retained in each of the final multi-variable models. Finally, bootstrapping was used to evaluate the robustness of selected proteins at 1000 iterations using the ‘BootValidation’ package in R on the elastic net model with the optimized regularization value (α=0.5) and analytes selected by the model for more than 60% ^65^ of times were considered as important protein features.

## Results

### Characteristics of study participants

Study participants’ characteristics are shown in Table 1. At enrolment, 89% of the children were over six months of age ^66^. Children were also severely stunted at enrolment and this was unchanged after 60 days despite large MUAC and weight gains with nutritional rehabilitation (all P.adj<0.01). Haemoglobin, total white blood cell count, and lymphocyte count increased, while neutrophil and platelet counts decreased between enrolment and 60 days (P.adj<0.01) ( Table 1).

**Table 1.** Characteristics of study participants.

Characteristic	Enrolment (N=98)	60 Days (N=98)	*P. _adj_*
**Demographics**			
Median age (mo.) [IQR]	13 [9–16]	–	–
Girls (n) %	47 (48)	–	–
Born prematurely (%)	14 (14)	–	–
Born underweight n (%)	23 (23)	–	–
**Recruitment hospital**			
Kilifi County Hospital n (%)	5 (5)	–	–
Coast General Hospital n (%)	51 (51)	–	–
Malindi Subcounty Hospital n (%)	20 (20)	–	–
Mbagathi County Hospital n (%)	24 (24)	–	–
**Randomized to co-trimoxazole n (%)**	50(50)		-
**Anthropometry**			
Weight (kg), mean ±SD	5.8±1.3	6.8±1.3	0.015
MUAC (cm), mean ±SD	10.6±1.0	11.9±1.1	<0.001
Height (cm), mean ±SD	66.8±7.3	68.8±6.9	<0.001
Weight absolute deficit (kg), mean ±SD	-3.2±1.1	-2.8±1.2	0.16
MUAC absolute deficit (cm), mean ±SD)	-3.8±0.9	-2.6±1.0	0.001
Height absolute deficit (cm) mean ±SD	-6.6±4.4	-7.2±4.3	0.012
WAZ, mean ±SD	-3.90±1.0	-3.07±1.2	0.011
WHZ, mean ±SD	-3.14±1.2	-1.85±1.4	0.016
HAZ, mean ±SD	-2.87±1.7	-3.02±1.5	0.001
**Full blood count**			
Haemoglobin g/dl mean ±SD	9.95±2.0	10.4±2.3	<0.001
WBC count (x10 ^3^/L) – median (IQR)	9.9 (6.3–12.7)	9.5 (6.6–12.1)	<0.001
Lymphocyte count (x10 ^3^/L) – median (IQR)	5.0 (2.9–6.7)	4.9 (3.0–7.2)	0.003
Neutrophil count (x10 ^3^/L) – median (IQR)	2.95 (1.9–4.7)	2.7 (2.1–4.3)	<0.001
Platelet count (x10 ^3^/L) – median (IQR)	475 (280–579)	407 (233–529)	<0.001

mo. = months, n = number of study participants, SD = standard deviation, IQR = interquartile range, P.adj = P value adjusted for age, sex, randomisation arm, and the site of enrolment, MUAC = mid-upper-arm circumference, WAZ = weight for age z score, WHZ = weight for height z score, HAZ = height for age z score, WBC = white blood cell.

### Children have higher growth rates during the first two months post-discharge

Overall, mean weight gain for 60 days was 3.3g (SD: ±2.4) per kilogram per day and 3.2kg (SD: ±0.27) during one year from enrolment. The mean MUAC and length/height gains for 60 days were 0.22mm (SD: ±0.2) per day and 0.34mm (SD: ±0.25) per day, respectively ( Table 2). The mean one-year gains in MUAC and height were 2.82cm (SD: ±1.34) and 11.15cm (SD: ±3.82), respectively. Changes in weight and MUAC from enrolment to 60 days were larger than during the later bimonthly time periods up to one year (p<0.01) ( Table 2). Differences in height between enrolment to 60 days were not significantly different from bimonthly changes up to 6 months (both p>0.1) but was higher than in bimonthly periods after 6 months ( Table 2).

**Table 2.** Bimonthly anthropometric growth indices of children during the first 180 days post-hospital discharge. * *p values* refer to paired t tests between changes during 0-60 days, and changes during 61-120 days, 121-180 days or average bimonthly changes between days 181 and 365. Enr.=Enrolment, Δ=change, DWAD=change in absolute deficits in weight, DMAD= change in absolute deficits in MUAC, DHAD=change in absolute deficits in height.

Changes in anthropometry								
Characteristic	Enr.–60 days	61–120 days	p value *	121–180 days	p value *	Bimonthly 181–365 days	p value *	Overall growth (Enr.–356 days)
Δ Weight (kg), mean ±SD	1.08±0.70	0.58±0.50	<0.001	0.40±0.44	<0.001	0.39±0.27	<0.001	3.23±0.27
Δ MUAC (cm), mean ±SD	1.33±0.89	0.51±0.65	<0.001	0.27±0.58	<0.001	0.24±0.29	<0.001	2.82±1.34
Δ Height (cm), mean ±SD	2.07±1.55	2.23±1.30	0.33	1.97±1.19	0.55	1.60±0.63	0.001	11.15±3.82
DWAD (kg), mean ±SD	-0.50±0.69	-0.10±0.48	<0.001	-0.05±0.43	<0.001	-0.02±0.28	<0.001	-0.51±1.23
DMAD (cm), mean ±SD	-1.20±0.89	-0.39±0.66	<0.001	-0.18±0.58	<0.001	-0.13±0.29	<0.001	-2.15±1.23
DHAD (cm), mean ±SD	-0.53±1.40	-0.08±1.23	0.03	-0.18±1.12	0.08	-0.29±0.59	0.37	-1.58±2.89

The mean change in absolute deficits in weight (DWAD) and MUAC (DMAD) from enrolment to 60 days were -0.5kg (SD: ±0.69) and -1.20cm (SD: ±0.89), respectively, and these were higher when compared to later time periods (P<0.001). There was a significant difference in the change in height deficit (DHAD) between the first 60 days and 61–120 days (P=0.03) but not at 121–180 days or the later bimonthly average change after 180 days (P>0.05). 

### Inflammatory cytokines and proteins are negatively associated with change in growth deficit at two months

**Figure 1.**
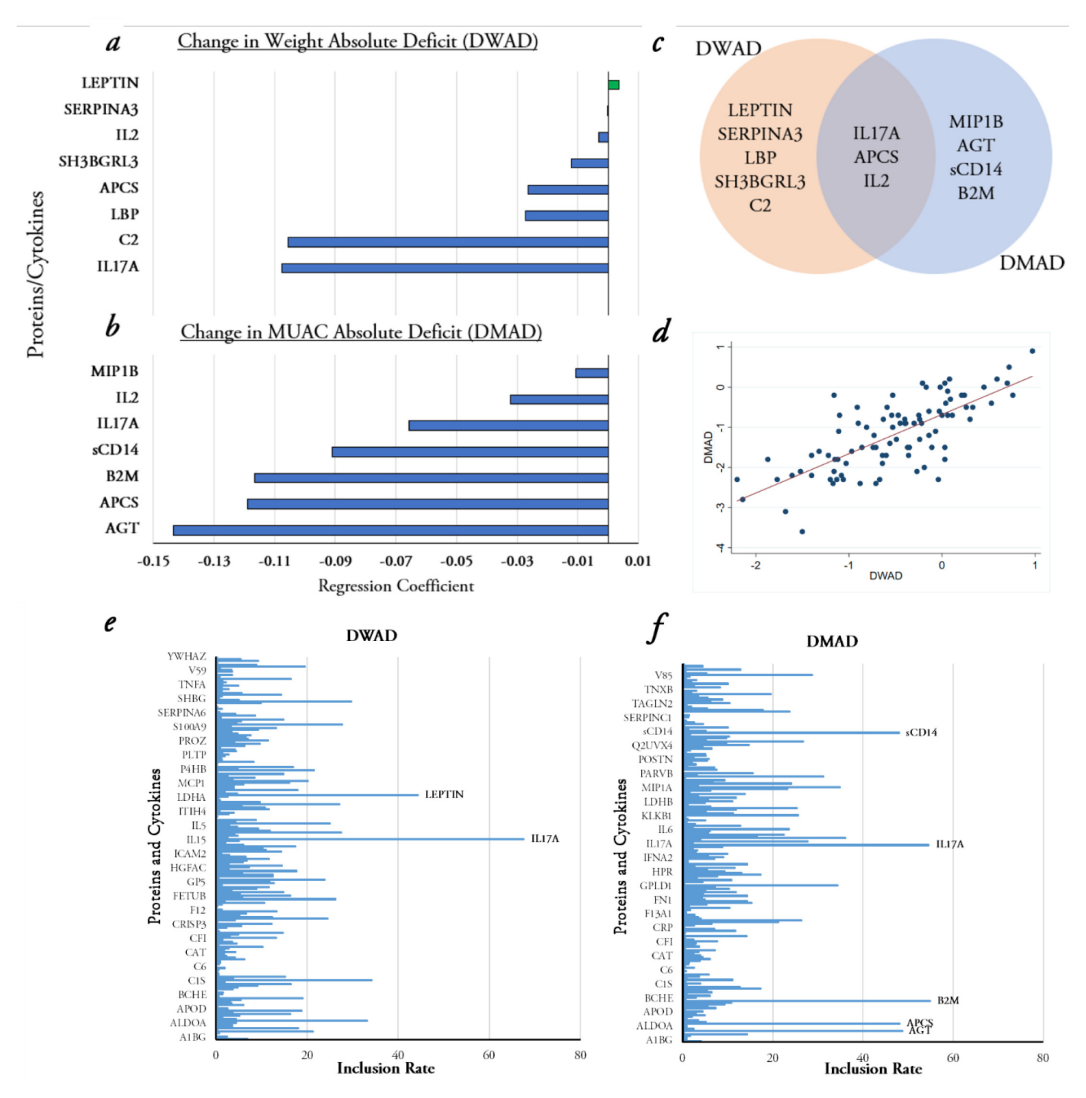
Multivariate analysis of plasma proteome and cytokines associated change in growth deficit at 60 days. Untargeted liquid chromatography tandem mass spectrometry plasma proteins, and targeted cytokines/chemokines, Leptin, and sCD14 associated with DWAD ( ***a***) and DMAD ( ***b***) in multivariate elastic net (EN) regularized linear regression models at two months. Log normalised protein values were used in the analysis and regression models were adjusted for age, randomisation arm, sex, respective enrolment growth deficits, and site. ( ***c***) A Venn diagram showing overlap of the proteins and cytokines associated with DWAD and DMAD. ( ***d***) A scatter plot showing that DWAD and DMAD are significantly correlated (P<0.001, R ^2^ =0.74). ( ***e*** and ***f***) Bar plots showing feature importance as depicted by the feature inclusion rate after 1000 bootstrap iterations during bootstrap validation for DWAD and DMAD, respectively. DWAD = change in weight absolute deficit, DMAD = change in MUAC absolute deficit, MUAC = mid-upper-arm circumference.

***Change in weight absolute deficit (DWAD)*.** In the multivariate elastic net (EN) regularized regression model adjusted for confounders, inflammatory cytokines interleukin 17 alpha (IL17a) and interleukin 2 (IL2), complement component 2 (C2), lipopolysaccharide binding protein (LBP), amyloid P component, serum (APCS or SAP), among others were negatively associated with DWAD in the first 60 days ( Figure 1a). Further, our analysis showed that the adipokine leptin was positively associated with DWAD ( Figure 1a).

***Change in MUAC absolute deficit (DMAD)*.** Inflammatory cytokines IL17a, IL2, and Macrophage inflammatory protein 1-beta (MIP1B) were negatively associated with DMAD in the first 60 days ( Figure 1b). Angiotensinogen (AGT), the precursor of all angiotensin peptides; soluble CD14 (sCD14), a co-receptor for the detection of bacterial lipopolysaccharide (LPS); beta-2 microglobulin (β2M), a component of MHC class I molecules which are present on all nucleated cells; and SAP, were negatively associated with DMAD ( Figure 1b). Only IL17a, IL2, and SAP were associated with both DWAD and DMAD ( Figure 1c) even though these two anthropometric measurements were significantly correlated as shown in Figure 1d. Both models were significantly associated to their respective growth outcome, accounting for just over half of the variability in growth (DWAD r ^2^=0.51 and DMAD r ^2^=0.57, Table 3).

**Table 3.** Elastic Net regression model optimal alpha parameters and performance of proteins associated with change in growth deficits within 60 days.

EN Variable		Optimal alpha	r	[95% CI]	P value
DWAD	Exposure protein variables	0.5	0.51	0.34 – 0.64	<0.0001
DMAD	Exposure protein variables	0.5	0.57	0.41 – 0.69	<0.0001

Footnote: Optimal alpha parameter and correlation coefficients for the EN model enumerating the correlation between DWAD and DMAD at two months and exposure protein variables (untargeted plasma proteome, and targeted cytokine/chemokines, leptin, and sCD14) extracted by the multivariate regularized models. EN = elastic net, DWAD = change in absolute deficits in weight, DMAD = change in absolute deficits in mid-upper-arm circumference, CI = confidence interval.

***Bootstrap analysis*.** After 1000 bootstrap iterations, only IL17a was identified in >60% of the DWAD model repetitions ( Figure 1e) indicating that this was the most robust feature associated with weight gain. Using similar iterations during bootstrap validation for the DMAD model, no features were extracted at the 60% threshold and the most frequently selected features were IL17a (55%), B2M (55%), AGT (49%), SAP (48%), and sCD14 (48%) as shown ( Figure 1f).

### Complement factors and Glutathione Peroxidase 3 are associated with one-year change in growth deficits

**Figure 2.**
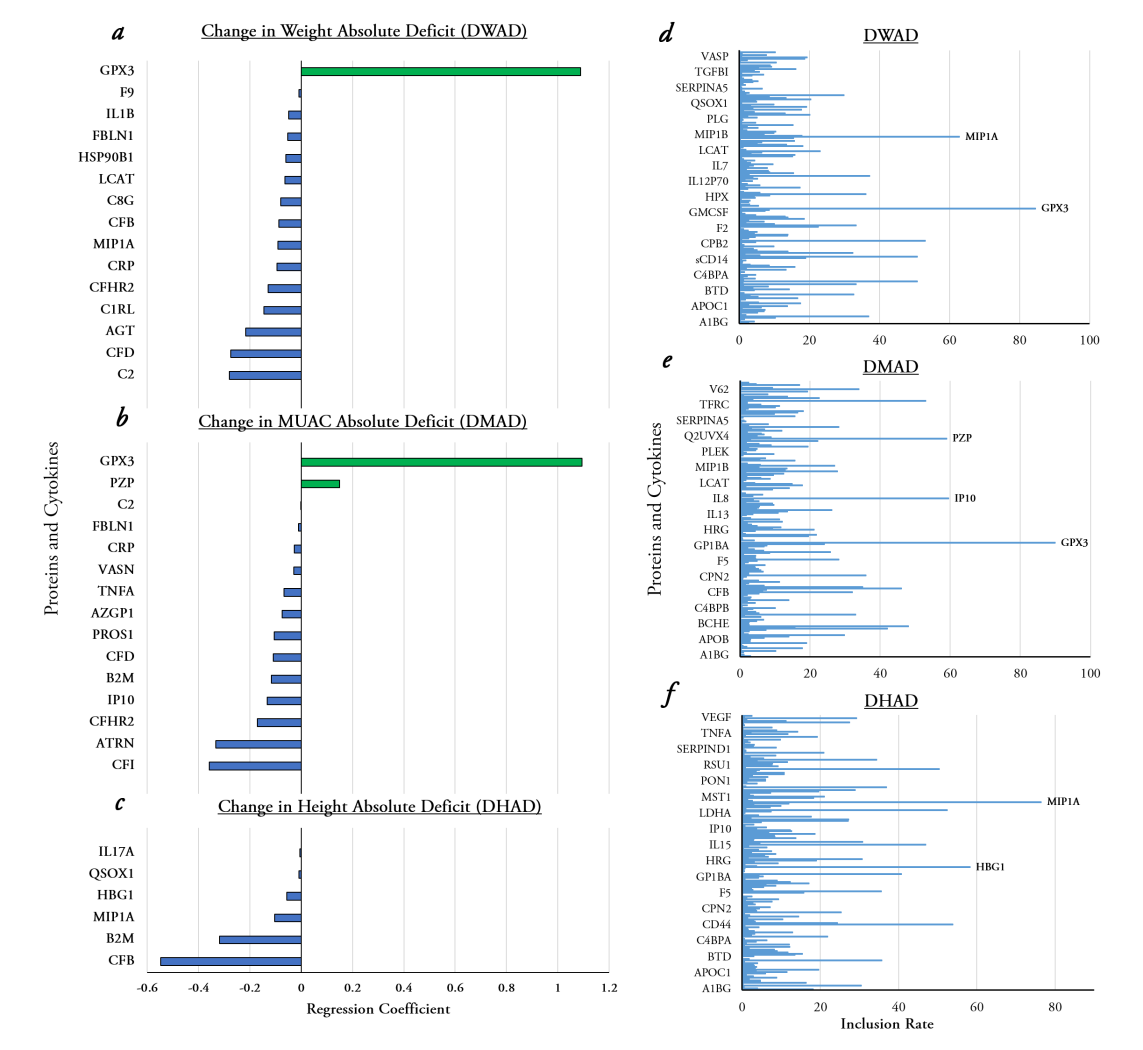
Multivariate analysis of plasma proteome and cytokines associated change in growth deficits from enrolment to one-year. Untargeted liquid chromatography tandem mass spectrometry plasma proteins, and targeted cytokines/chemokines, Leptin, and sCD14 associated with DWAD ( ***a***), DMAD ( ***b***) and DHAD ( ***c***) in multivariate elastic net (EN) regularized linear regression models at 1 year. ( ***d***, ***e***, and ***f ***) Bar plots showing feature importance as depicted by the feature inclusion rate after 1000 bootstrap iterations during bootstrap validation for DWAD, DMAD, and DHAD respectively. Log normalised protein values were used in the analysis and regression models were adjusted for age, randomisation arm, sex, respective enrolment growth deficits, and site.

***DWAD at one-year follow-up.*** Complement proteins; C2, factor D (CFD), C1r Subcomponent Like (C1RL), factor B (CFB), and component 8 (C8G), as well as AGT, CRP, and cytokines; MIP1a and IL1b were negatively associated with DWAD from enrolment to one year ( Figure 2a). Glutathione Peroxidase 3 (GPX3), a selenium-dependent antioxidant enzyme that scavenge hydrogen peroxide in the presence of reduced glutathione was positively associated with one-year DWAD.

***DMAD at one-year follow-up.*** Complement proteins; factor I (CFI), factor H Related 2 (CFHR2), CFD, and C2, as well as Attractin (ATRN), β2M, CRP, Zinc-alpha-2-glycoprotein (AZGP1 or ZAG), Vitamin K-dependent protein S (PROS1), and cytokines; IP10 and TNFα were negatively associated with one-year DMAD ( Figure 2b). GPX3 and Pregnancy zone protein (PZP Alpha-2-Macroglobulin Like) was positively associated with one-year DWAD ( Figure 2b).

***DHAD at one-year follow-up.*** Complement factor B (CFB), β2M, MIP1a, and Haemoglobin Subunit Gamma 1 (HBG1) among others were negatively associated with one-year DHAD.

***Bootstrap analysis (enrolment one–year).*** Using 1000 bootstrap iterations, GPX3 and MIP1a were identified in >60% of the one-year DWAD model repetitions ( Figure 2d). GPX3 was also selected at >60% in the bootstrap validation for the DMAD model. Other frequently selected proteins included IP10 (60%) and PZP (59%). MIP1a was selected at >60% in the bootstrap validation of DHAD while HBG1 was selected at 58% of the iterations. 

All the models were significantly associated to their respective growth outcome, accounting for over half of the variability in growth ( Table 4).

**Table 4.** Elastic Net regression model optimal alpha parameters and performance of proteins associated with change in growth deficits at one-year.

EN Variable		Optimal alpha	r	[95% CI]	P value
DWAD	Exposure protein variables	0.5	0.73	0.62 – 0.82	<0.0001
DMAD	Exposure protein variables	0.5	0.72	0.59 – 0.81	<0.0001
DHAD	Exposure protein variables	0.5	0.58	0.42 – 0.71	<0.0001

**Footnote:** Optimal alpha parameter and correlation coefficients for the EN model enumerating the correlation between DWAD, DMAD, and DHAD at one-year and exposure protein variables (untargeted plasma proteome, and targeted cytokine/chemokines, leptin, and sCD14) extracted by the multivariate regularized models.

## Discussion

We investigated the relationship between inflammatory cytokines and plasma proteomic profiles and change in anthropometric deficits during the early post-hospital discharge period as this is the period most likely to be related to biological factors measured at discharge and when catch up in weight deficit is at its greatest ^8^. We also investigated associations between discharge proteomic profiles and inflammation status and change in growth deficits over one-year follow-up since little is known about extended influences of inflammation and longer-term growth in acutely ill undernourished populations. The mean weight gain rate of 3.3 g/kg/day observed was comparable to that reported for uncomplicated SM treated with a similar diet in the community ^31, 67– 69^. However, there were significant reductions in absolute deficits of weight and MUAC. Although markers of SI were negatively associated with growth in the early post-hospital discharge period, substantial growth did occur in the presence of markers of inflammation. It is likely that the large metabolic shifts observed during refeeding with energy dense therapeutic feeds favours tissue accretion even in the presence of SI. It is notable that despite absolute increases in height, there was no significant reduction in absolute deficit of height, indicating that catch-up growth mainly occurs in the adipose and muscle and not long bones. No comparable data on inflammatory markers are published from children with uncomplicated SM. However, lack of an acute illness means it is plausible that there is less systemic inflammation (SI). Overall, our results indicate that growth is influenced by inflammation status.

Inflammatory cytokines IL17a, IL2, and MIP1B and inflammatory proteins sCD14, LBP, SAP, and β2M were negatively associated with weight gain and MUAC in the early post-discharge period. IL17a is produced by T-helper 17 (Th17) cells that play a role in host defence against extracellular pathogens through recruitment of neutrophils and macrophages to infected tissues ^70– 72^. IL17a ^73^ is involved in tissue inflammation by release of other pro-inflammatory cytokines and inducing neutrophil chemotaxis ^74^ and is implicated in obesity and adipogenesis ^75^. In mouse models, IL17a has been proposed to play a role in weight gain in response to a high-fat diet ^76^. In humans, increased expression of IL17a has been reported in inflammatory bowel disease ^77– 79^. sCD14 is secreted by monocytes and macrophages commonly in response to LPS translocation ^80^, while LBP is a plasma protein that binds to the lipid A moiety of bacterial LPS ^81^. β2M is released by activated T and B lymphocytes and plasma β2M has been described as a predictive biomarker for many vascular inflammatory diseases ^82, 83^. SAP is an acute phase protein and belongs to the pentraxins family of proteins that also includes C-reactive protein, which exhibit calcium-dependent binding to several different molecules and pathogens ^84^. Children included in this study were judged by trained clinicians as clinically stabilised following an acute illness and induction of these inflammatory cytokines and proteins likely results from induction by microbial molecules ^85, 86^. Several of these cytokines are elevated in patients with inflammatory bowel disease ^77, 87– 89^ and may reflect the presence of environmental enteric dysfunction, which is common in low- and middle-income countries, that is associated with linear growth failure ^51, 90, 91^.

Complement proteins were negatively associated with one-year gains in weight, MUAC and height. The complement system is comprised of plasma proteins and is part of the innate defence against common pathogens that enhances the ability of antibodies and phagocytic cells to opsonize and lyse microbes and damaged cells and promote inflammation ^92^. The complement system is activated via three different pathways: the classic pathway, the alternative pathway, and the lectin pathway and the adipose tissue is the site of production of some complement components ^93^. The activation of the alternative pathway is composed of complement C3, factors B and D which are mainly produced by the adipose tissue ^94– 96^. Previous studies have shown that levels of serum complement proteins are highly correlated with body weight and changes in body weight ^97, 98^. Malnutrition has been associated with low complement levels ^99^, although studied populations may have had concurrent infections or conditions which might affect complement levels. The negative association between complement and one-year growth implies that inflammation at discharge likely impacts longer-term growth in this population. 

The negative relationship between growth at one year and inflammation as depicted by CRP, Attractin (ATRN; expressed on activated T cells and released extracellularly) and cytokines, mirrored findings from the early post discharge growth which was the period with the highest growth rate. We also found that Zinc-alpha-2-glycoprotein (ZAG) was negatively associated with gain in MUAC. ZAG also known as lipid-mobilizing factor stimulates lipolysis and is involved in depletion of fatty acids from adipose tissues ^100– 102^.

Our results show that leptin was positively associated with early post-discharge growth. Leptin levels increase with accretion of adipose tissue mass and therefore leptin levels are related to body weight ^103^. Leptin was first recognized for its prominent action on the hypothalamus to control food intake, energy expenditure and, hence, body weight ^104– 106^. It is also involved in immune homeostasis by differentially regulating T cells, enhancing Th1 and suppressing Th2 cytokine production, and reversing starvation-induced immunosuppression ^107– 109^. Among Ugandan children hospitalised with SM, nutritional stabilisation and weight gain was associated with significant increases in leptin levels ^57^. However, it is worth noting that leptin levels which were measured at hospital discharge may still be low among children with SM.

One-year gains in weight and MUAC were positively associated with GPX3 at enrolment. GPX3 is a selenium-dependent enzyme playing a critical role in detoxifying reactive oxidative species including organic and lipid hydroperoxides as well as hydrogen peroxide ^110– 112^. Selenium via selenoproteins plays a crucial role in cellular redox balance, immunity, and metabolism and GPX3 is a marker of differentiated adipocytes ^113, 114^. In animal models, adipose GPX3 expression was suppressed by TNFα, LPS-induced SI, and by hypoxia, but was stimulated by the antioxidant N-acetyl cysteine ^114^. Recent findings implicate GPX3 as a regulator of insulin receptor expression and insulin sensitivity in adipose tissue ^115^. GPX3 has been identified as one of the strongest genes associated with traits of insulin sensitivity, adipogenesis, and Type 2 Diabetes ^116^. GPX3 also belongs to a cluster of adipokines which is closely related to insulin sensitivity, hyperglycemia, and lipid metabolism ^117^. Taken together, our findings imply that oxidative stress and increased SI during the acute phase are pathways that contribute to long-term post-discharge growth failure among acutely ill undernourished children.

Proinflammatory signalling in the adipocyte is required for proper adipose tissue remodelling and expansion ^118^ and recent studies suggest that low grade inflammation may play a positive role in weight gain in both children ^119^ and adults ^120^. In a population-based longitudinal study in the Brazilian Amazon among children ≤10 years, low-grade inflammation (CRP <1 mg/L at baseline) was predictive of annual gain in BMI-for-age during follow-up ^119^. During refeeding, severely malnourished children may adopt an obesogenic metabolic phenotype, where tissue accretion occurs in the presence of inflammation and reflecting restoration of adipose tissue lost due to malnutrition ^121^. However, inflammation increases energy expenditure and in animal studies focusing on increasing production, SI is attributed to inefficient nutrient utilization efficiency which translated to low gain in weight, implying that in that context, persistent inflammation negatively affects growth ^122– 125^. However, although we previously found CRP to be associated with mortality ^37^, other biomarkers of inflammation were negatively associated with growth in the present study.

Future work should investigate effects over a longer time period post-discharge and compare with community participants/children without acute illness to establish community norms and examine interventions to reduce inflammation. The strengths of this study included robust and standardised data collection within a clinical trial, follow-up for one year and very low loss to follow-up. The limitations include the relatively small sample size, the lack of serial measurements of inflammatory markers and body composition, the inability to control for other factors that may influence cytokine levels, and that children with oedema were excluded from the analysis. This study was carried out at four sites in Kenya only. Every child was tested for HIV, enabling us to exclude its effect. Important nutritional factors, hormones and growth factors, and metabolites which would have contributed further to the understanding of the relationship between SI and growth were not determined. Molecules such as LPS that would explain elevated SI were not determined and were beyond the scope of this study. The untargeted proteomics and targeted Luminex and ELISA approaches used in this study provided a broad array of protein molecules from which to identify molecules associated with early post-hospital discharge growth.

## Conclusions

Among children with SM, early post-hospital discharge catch-up growth in weight and MUAC is rapid. Higher concentrations of markers of SI were associated with less early weight and MUAC gain, suggesting inflammation negatively impacts recovery from wasting. Our results indicate that growth is influenced by inflammation status and warrants further research on the role of inflammation on growth among children with SM.

## Data availability

### Underlying data

Specific variables such as personal identifiers and the longitude and latitude co-ordinates of study participants were removed to enhance participant anonymisation and can be accessed following application to our Data Governance Committee at dgc@kemri-wellcome.org. The replication data and analysis scripts for this manuscript are available from the Harvard Dataverse.

Harvard Dataverse: Replication data for: Systemic Inflammation is Negatively Associated with Early Post Discharge Growth following Acute Illness among Severely Malnourished Children- a Pilot Study. https://doi.org/10.7910/DVN/5DKLVI ^66^.

This project contains the following underlying data:

- (a) Njunge_CTX_15092020.dta
- (b) Njunge_CTX_15092020.csv

Both (a) and (b) files contain similar information. The files contain anthropometric measurements at the time of hospital discharge and during follow up months 1, 2, 3, 4, 5, 6, 8, 10, and 12. A full blood count at enrolment, 2, 6, and 12 months. The two files were generated using STATA/IC (version 15.1; StataCorp, College Station, TX, USA)

- Njunge_Inflammation_Codebook.pdf: It contains a list of all the variables in the two datasets and their description.

### Extended data

Harvard Dataverse: Replication data for: Systemic Inflammation is Negatively Associated with Early Post Discharge Growth following Acute Illness among Severely Malnourished Children- a Pilot Study. https://doi.org/10.7910/DVN/5DKLVI ^66^.

This project contains the following extended data:

- Njunge_EN_Glmnet_bootValidation.R: This analysis script uses Njunge_CTX_15092020.csv to perform multivariate regularized linear regression analysis using an elastic net (EN) model implemented using the “glmnet” package and fits a generalized linear model via penalized maximum likelihood. It generates an EN model separately for each growth outcome (change in Weight Absolute Deficit(DWAD) (primary outcome) and change in MUAC Absolute Deficit (DMAD) (secondary outcome), with protein profiles, cytokines, and enrolment anthropometric variables as predictors. The subset of variables assigned non-zero coefficients are retained in each of the final multi-variable models. It then performs Bootstrapping to evaluate the robustness of selected proteins at 1000 iterations using the ‘BootValidation’ package. The analysis was performed using R Studio (R version 3.6.2 (2019-12-12))
- Njunge_Stata_Do.do: This analysis script uses Njunge_CTX_15092020.dta to generate the summary participants characteristics at enrolment and at 2 months and calculate the changes in anthropometry
- NjungeJM_Inflammation_Readme.txt: It contains description of the related study, file contents, data license and usage instructions.

Data are available under the terms of the Creative Commons Attribution 4.0 International license (CC-BY 4.0).
