# Supplementary material for: Systemic inflammation is negatively associated with early post discharge growth following acute illness among severely malnourished children - a pilot study
Source: Wellcome Open Res. 2021 Mar 16;5:248. Originally published 2020 Oct 22. [Version 2] doi: 10.12688/wellcomeopenres.16330.2 (PMC8080977; doi:10.12688/wellcomeopenres.16330.2)
Supplement: Peer review history

# Peer review: Reviewer response for version 2

**DOI:** 10.21956/wellcomeopenres.18425.r43091

**Published:** 2021-04-23

**Authors:** Sana Syed, Lubaina Ehsan

**Affiliations:** 1 University of Virginia, Charlottesville, VA, USA; 2 Division of Pediatric Gastroenterology, Hepatology and Nutrition, Department of Pediatrics, School of Medicine, University of Virginia, Charlottesville, VA, USA

The authors have responded to all my concerns. I have no further comment and approve this manuscript.

Is the work clearly and accurately presented and does it cite the current literature?

Yes

If applicable, is the statistical analysis and its interpretation appropriate?

Yes

Are all the source data underlying the results available to ensure full reproducibility?

Partly

Is the study design appropriate and is the work technically sound?

Yes

Are the conclusions drawn adequately supported by the results?

Yes

Are sufficient details of methods and analysis provided to allow replication by others?

Yes

Reviewer Expertise:

Gastroenterology, Nutrition, Data Science

We confirm that we have read this submission and believe that we have an appropriate level of expertise to confirm that it is of an acceptable scientific standard.

# Peer review: Reviewer response for version 2

**DOI:** 10.21956/wellcomeopenres.18425.r43090

**Published:** 2021-03-22

**Authors:** Keith P. West

**Affiliations:** 1 Johns Hopkins University Bloomberg School of Public Health, Baltimore, MD, USA

The paper reads well and the authors have responded fully to my concerns. I have no further comments and approve the manuscript. A minor query that does not affect my approval is that the 1st “D” in the acronyms DWAD, DMAD and DHAD presumably refers to “delta”, but this is not explicit. As each is defined as a “change” in weight, MUAC or height, absolute difference, might these be respecified to CWAD, CMAD and CHAD? Alternatively, I may have also missed something and nothing needs to be altered.

Is the work clearly and accurately presented and does it cite the current literature?

Yes

If applicable, is the statistical analysis and its interpretation appropriate?

I cannot comment. A qualified statistician is required.

Are all the source data underlying the results available to ensure full reproducibility?

Yes

Is the study design appropriate and is the work technically sound?

Yes

Are the conclusions drawn adequately supported by the results?

Yes

Are sufficient details of methods and analysis provided to allow replication by others?

Partly

Reviewer Expertise:

Nuritional epidemiology, applied proteomics, nutrition interventions, child growth, maternal nutrition.

I confirm that I have read this submission and believe that I have an appropriate level of expertise to confirm that it is of an acceptable scientific standard.

# Peer review: Reviewer response for version 1

**DOI:** 10.21956/wellcomeopenres.17953.r41116

**Published:** 2020-12-09

**Authors:** Keith P. West

**Affiliations:** 1 Johns Hopkins University Bloomberg School of Public Health, Baltimore, MD, USA

This study examines the potential attenuating influence of inflammation at time of discharge on subsequent linear and ponderal growth of young children initially hospitalized for severe complicated, non-HIV-related malnutrition.  Inflammation at discharge was assessed by a combined assessment of cytokines (IL-2, IL17a and SAP) and relative abundance of plasma protein biomarkers, including those whose expression covaries with phases of the inflammatory response.  The study is an important follow-up from a previous case-control study (Njunge et al Sci Reports 2019 ^1^) but also because it adds new insights amidst a sparsity of studies harnessing the potential of plasma proteomics to reveal metabolic alterations and pathways that may be affecting recovery-related (catch-up) child growth following severe malnutrition.

 Comments:

 The methods would benefit from slightly more essential study details that can render this paper independent of a previous paper (Njunge et al., Sci Reports 2019 ^1^) as I found myself needing to return to that publication to better understand aspects of study design:  please clarify when children were phlebotomized (presumably just prior to discharge that constitute enrolment samples), which is also equivalent to baseline. 

 Notwithstanding important details provided about the analytical methods, thresholds at which associations between DMAD and DWAD and protein relative abundance/cytokine concentrations are considered chance-adjusted, statistically significant are not specified. Is there a p- or q-value considered to be SS, and how were family-wise error probabilities from multiple comparisons managed? P-values for EN models for DWAD and DMAD are given in Table 3 but its not clear what these are testing or how derived.

 Children were followed monthly through 6 mo and bi-monthly thereafter to 12 months. Given the measurements are available through 1-year post discharge, and partly reported as 60-day increments to 6 months of age in Table 2, the rationale for restricting evaluation of biomarker-growth deficit recovery to the 1 ^st^ 60 days of follow-up, as opposed to longer, would benefit from clearer argument. It would be easier to follow if all data were restricted to a 60-day period for this paper, which aligns with the survival findings reported in Njunge et al 2019 ^1^. That said, reporting associations between discharge inflammation status and growth over longer intervals would be an important contribution, as little is known about extended influences of inflammation and longer term growth in undernourished populations. One population cohort-based plasma proteomics study in Nepal has shown head size at birth (Lee SE et al Sci Reports 2018 ^2^) and attained HAZ and WAZ at 6-8 years of age (Lee SE et al J Nutr 2017 ^3^), to be associated with the relative abundance of a wide array of plasma proteins, including S100 calprotectins (A9 and A12 isomers, respectively), assessed at 6-8 years. The few such studies that exist suggest persistent (long term), bidirectional associations between child growth and metabolic pathways, as expressed through the plasma proteome, observations that pique interest to explore protein biomarker associations with extended growth endpoints, in this paper or a subsequent treatise.

 To the extent time points are maintained, can the authors clarify if children were measured at exactly 60, 120 and 180 days post-enrollment?  If not, how were growth intervals standardized to 60-day periods?  

 DHAD data are summarized in Table 2. Why was it not also a primary outcome?

 Table 1: In the 1 ^st^ row, it would be informative to have a summary [median (IQR)] of ages of children at 60 days (removing “at enrolment” in the row label). 

 Table 3: The title is long and difficult to understand. Mention is made of correlation, but R ^2^ is reported. Please shorten and articulate specifics in the footnotes, including the exposure protein variables in each model (assuming these are the proteins reported in Figure 1 and b?).

 Figure 1: Please clarify the x-axis label in a and b as regression coefficient and in the legend, specify measurement unit for each. Findings from Figures 1e and 1f, presenting Inclusion Rates associated with DWAD and DMAD, are presently not mentioned in the Results. 

                

 Discussion

 Three-quarters down the 1 ^st^ paragraph, noting the height deficit did not show evidence of recovery, could this be placing too high an expectation on long bone growth recovery commensurate with regaining soft tissue accretion within only 60 days?

 Few typos: 

 Figure 1: Within the parentheses (d and f) should be (e and f);

 In discussion – LBP is a plasma protein that binds...

Is the work clearly and accurately presented and does it cite the current literature?

Yes

If applicable, is the statistical analysis and its interpretation appropriate?

I cannot comment. A qualified statistician is required.

Are all the source data underlying the results available to ensure full reproducibility?

Yes

Is the study design appropriate and is the work technically sound?

Yes

Are the conclusions drawn adequately supported by the results?

Yes

Are sufficient details of methods and analysis provided to allow replication by others?

Partly

Reviewer Expertise:

Nuritional epidemiology, applied proteomics, nutrition interventions, child growth, maternal nutrition.

I confirm that I have read this submission and believe that I have an appropriate level of expertise to confirm that it is of an acceptable scientific standard, however I have significant reservations, as outlined above.

## References

[1] : Biomarkers of post-discharge mortality among children with complicated severe acute malnutrition. *Scientific Reports*.2019;9(1) : 10.1038/s41598-019-42436-y 10.1038/s41598-019-42436-y 30979939PMC6461700

[2] : Novel Plasma Proteins in Nepalese School-aged Children are Associated with a Small Head Size at Birth. *Scientific Reports*.2018;8(1) : 10.1038/s41598-018-24640-4 10.1038/s41598-018-24640-4 29686285PMC5913316

[3] : The Plasma Proteome Is Associated with Anthropometric Status of Undernourished Nepalese School-Aged Children. *J Nutr*.147(3) : 10.3945/jn.116.243014 304-313 10.3945/jn.116.243014 28148680PMC5320403

# Response

**Published:** 2021-03-05

**Authors:** James Njunge

**Affiliations:** KEMRI-Wellcome Trust, Kenya

**Reviewer 1**

**Sana Syed, Division of Pediatric Gastroenterology, Hepatology and Nutrition, Department of Pediatrics, School of Medicine, University of Virginia, Charlottesville, VA, USA.**

**Lubaina Ehsan, University of Virginia, Charlottesville, VA, USA**

**Approved With Reservations**

**The manuscript submitted by Dr. Njunge and colleagues aims to investigate the association of systemic inflammation among early post-discharge children who are severely malnourished and have had an acute illness. Overall, this manuscript presents results that would be of interest to the community of scientists and clinicians concerned with this problem, major strengths of their work are: **

- **Well-written and coherent manuscript which clearly outlines the work that was done.**
- **Strong statistical analysis of not only the weight/MUAC gain but also the absolute deficits in these measurements.**
- **A discussion that outlines each significant result clearly with extensive literature being referenced.**
- **The assessment of potential markers for growth being associated with inflammation which can pave the way for revised standards of anthropometric measurement among children within the same cohort.**

**However, there are a few issues in this manuscript that prevent us from recommending that this manuscript be indexed in its current state:**

****

**Overall**

**There is a mismatch between the introduction and the discussion. While the introduction mentions RUTF and GH/IGF1 axis, we also suggest adding the background behind the assessment of the relationship of plasma proteomic and cytokine profiles with growth. The discussion then excellently discussed the individual results for cytokines and other markers although, the background for these measurements being part of the introduction will make the foundation of why this work was done even more strong. **

- Studies detailing the relationship between plasma proteomics and cytokine profiles on catch-up growth after an acute illness are very limited among undernourished children. We have added additional citations on linear growth. “ *In Nepal, birth size was inversely associated with low-grade, chronic inflammation during pregnancy as indicated by serum AGP* *^1,2^* *. Further, head size at birth among Nepalese* *^3^* *and attained z scores for height (HAZ) and weight-for-age (WAZ) at 6-8 years of age* *^4^* *were associated with a wide array of plasma proteins, including S100 calprotectin subunits, assessed at 6-8 years.* ** *In* *community settings, SI is* *elevated in 17-34% children in* *LMIC* *^5^* *(C-reactive protein >5mg/L or α1-antichymotrypsin >0.6g/L) and is associated with reduced linear growth* *^6-10^* *.* *”* However the literature is quite limited ^11^.

- **The proteomics and cytokines measurements in plasma were measured using liquid chromatography tandem mass spectrometry and targeted cytokines, chemokines, leptin and soluble CD14 by Luminex and ELISA; however, cytokines are known to be variable and fluctuate in different physiological locations and environments, which is a limitation of the study. We would suggest the authors describe how they overcame this limitation. **
  We thank the reviewers for pointing out this limitation which we highlight in our study. We have added a statement in the main text about other unmeasured factors causing fluctuations in cytokine levels *“we were unable to control for other factors that may influence cytokine levels”.* Further, we lacked serial measurements of inflammatory markers. However, it has been shown that “ *in high income settings, survivors of sepsis have elevated markers of SI for up to 1 year and retain an increased mortality risk* *^12-16^* *. After sepsis, systemic levels of C-reactive protein and soluble programmed death ligand 1 (a marker of immunosuppression) are elevated for up to 1 year in patients while IL6 and IL10 persists for several months in human and experimental models* *^12-14^* *.”* These data implicate SI in both short and longer-term growth outcomes post-discharge. We also agree with Reviewer 2 who points out that “reporting associations between discharge inflammation status and growth over longer intervals would be an important contribution, as little is known about extended influences of inflammation and longer-term growth in undernourished populations”.

**Methods**

- **It is mentioned that the data was from a nested case control study within a clinical trial (NCT00934492) that tested the efficacy of daily co-trimoxazole prophylaxis. However, there was no comment found on the potential effect of co-trimoxazole prophylaxis as a confounder on the results for systematic inflammation. We suggest that a short explanation is added, whether in the main manuscript or supplemental material.**
  This was conducted and is indicated under the bioinformatics and statistics section of the manuscript. We adjusted for randomisation in the regression models to account for the potential effect of co-trimoxazole prophylaxis.
- **The authors have used a 60-day cut-off post-discharge – was there a particular reason for selecting this and not any earlier/later time-point and any literature supporting assessment of growth at 60 days post-discharge?**
  We selected the 60-day post-discharge period because it’s when catch up in weight deficit was at its greatest and allows for short term convalescence. We also wished to retain the time-period at 60 days post-discharge as was the original design of the case-control mortality analysis ^17^. This is also the period most likely to be related to biological factors measured at discharge unlike the later time-points. Following the reviewers’ suggestions on the lack of data regarding extended influences of inflammation and longer-term growth in undernourished populations, we have conducted further analysis with growth deficits (weight, MUAC, and height) at 1 year and presented these results in the main text. We believe that this analysis on longer-term impact on growth is a valuable contribution to the field where the data is quite limited. Future analysis utilising other cohorts including the Childhood Acute Illness and Nutrition (CHAIN) Network Cohort in Kenya, Uganda, Malawi, Burkina Faso, Bangladesh and Pakistan that is examining mortality, readmission and growth among acutely ill children below 2 years ^18^ will focus on longer time periods post-discharge. This was a pilot study and there is no literature to the best of our knowledge that guides assessment of growth at 60 days post-discharge. However, most community-based feeding studies have assessed growth between 1 and 6 months ^19-26^.
- **We suggested clarifying how ‘absolute deficit’ was calculated. **Absolute deficit was defined as the median value for age according to WHO growth charts minus the child’s measured value. We have added text to indicate that *“It was calculated as the difference between the measured weight, height, or MUAC and the median age- and sex-specific value obtained from the WHO 2006 growth standards* *^27-29^* *”* *.*

**Results**

- **Was there a difference noted for patients that were born prematurely or underweight since the eventual weight gain may have been due to regression to the mean? Any analysis that was done after excluding these patients?**
  Regression to the mean was addressed by including enrolment anthropometric values in the regression models. Except for one child, all children born premature were also underweight. The model outcomes did not change when we included the underweight variable in the model.

**Discussion**

- **A few comments are mentioned under the ‘overall’ heading.**
**Any future directions for the current work?**
- Yes. “ *Future work should investigate effects over a longer time period post-discharge and compare with community participants/children without acute illness to establish community norms.*” We have added this to the main text.
- **We also briefly suggest adding strengths of the study, although not necessary.**
  Strengths included robust and standardised data collection within a clinical trial, follow-up for one year and very low loss to follow-up. This has been added on the main text.

**Reviewer 2.**

**Keith P. West** **,** **Johns Hopkins University Bloomberg School of Public Health, Baltimore, MD, USA**

**Approved With Reservations**

**This study examines the potential attenuating influence of inflammation at time of discharge on subsequent linear and ponderal growth of young children initially hospitalized for severe complicated, non-HIV-related malnutrition.  Inflammation at discharge was assessed by a combined assessment of cytokines (IL-2, IL17a and SAP) and relative abundance of plasma protein biomarkers, including those whose expression covaries with phases of the inflammatory response.  The study is an important follow-up from a previous case-control study (Njunge et al Sci Reports 2019 ^1^) but also because it adds new insights amidst a sparsity of studies harnessing the potential of plasma proteomics to reveal metabolic alterations and pathways that may be affecting recovery-related (catch-up) child growth following severe malnutrition.**

**Comments:**

**The methods would benefit from slightly more essential study details that can render this paper independent of a previous paper (Njunge et al., Sci Reports 2019 ^1^) as I found myself needing to return to that publication to better understand aspects of study design:  please clarify when children were phlebotomized (presumably just prior to discharge that constitute enrolment samples), which is also equivalent to baseline.**

 We have adjusted the text to indicate that *“Samples were collected from study participants prior to initiation of the investigational product: co-trimoxazole or placebo at discharge and constitute enrolment samples”.*

**Notwithstanding important details provided about the analytical methods, thresholds at which associations between DMAD and DWAD and protein relative abundance/cytokine concentrations are considered chance-adjusted, statistically significant are not specified. Is there a p- or q-value considered to be SS, and how were family-wise error probabilities from multiple comparisons managed? P-values for EN models for DWAD and DMAD are given in Table 3 but its not clear what these are testing or how derived.**

 We did not infer associations between DMAD or DWAD with individual proteins. Instead, we directly performed a penalised multivariate analysis to model the association between all proteins and growth outcomes. Hence, only 2 multivariate models were made, resulting to the p-values reported in Table 3. Adjustment for multiple comparison is not typically used for these types of models and the elastic net method utilised aggressively penalised redundant and non-predictive protein features. The p-values for the EN models in Table 3 are the association between the actual DWAD/DMAD to the predicted DWAD/DMAD of the model. Therefore, the r value therefore represents the variance explained by the model, in which case our models captured 51% and 57% of the variance for DWAD and DMAD at month two, respectively.

**Children were followed monthly through 6 mo and bi-monthly thereafter to 12 months. Given the measurements are available through 1-year post discharge, and partly reported as 60-day increments to 6 months of age in Table 2, the rationale for restricting evaluation of biomarker-growth deficit recovery to the 1 ^st^ 60 days of follow-up, as opposed to longer, would benefit from clearer argument. It would be easier to follow if all data were restricted to a 60-day period for this paper, which aligns with the survival findings reported in Njunge et al 2019 ^1^. That said, reporting associations between discharge inflammation status and growth over longer intervals would be an important contribution, as little is known about extended influences of inflammation and longer term growth in undernourished populations. One population cohort-based plasma proteomics study in Nepal has shown head size at birth (Lee SE et al Sci Reports 2018 ^2^) and attained HAZ and WAZ at 6-8 years of age (Lee SE et al J Nutr 2017 ^3^), to be associated with the relative abundance of a wide array of plasma proteins, including S100 calprotectins (A9 and A12 isomers, respectively), assessed at 6-8 years. The few such studies that exist suggest persistent (long term), bidirectional associations between child growth and metabolic pathways, as expressed through the plasma proteome, observations that pique interest to explore protein biomarker associations with extended growth endpoints, in this paper or a subsequent treatise.**

 We thank both reviewers for questioning the rationale for restricting evaluation of biomarker-growth deficit recovery to the 1 ^st^ 60 days of follow-up, as opposed to longer. We have addressed this under reviewer 1 comment 2 in the methods section. We agree that reporting associations between discharge inflammation status and growth over longer intervals would be an important contribution, as little is known about extended influences of inflammation and longer-term growth in undernourished populations. We have therefore conducted further analysis with changes in growth deficits (weight, MUAC, and height) during one-year from enrolment, presented the results in the main text and discussed the findings. An interesting and strong association between Glutathione peroxidase 3 and one-year weight and MUAC deficit changes emerged. To extend this work, our next step is a planned detailed longitudinal analysis of the relationship between systemic inflammation and growth outcomes post-discharge among acutely ill children right across the nutritional spectrum using data and samples from a large recently completed cohort study in Africa and Asia ^18^.

**To the extent time points are maintained, can the authors clarify if children were measured at exactly 60, 120 and 180 days post-enrollment?  If not, how were growth intervals standardized to 60-day periods?**

 The mean number of days from enrolment to month two was 61.7 days (SD; 1.98), median [IQR] was 61 [61-63] days. The mean (SD) and median [IQR] number of days between months 2 – 4 and months 4 – 6 were: 61.5 (3.40); 61 [60-63] and 61.5 (6.41); 61 [59-63] respectively. Calculations on growth velocity were based on the difference between the measured values at enrolment and month two divided by the difference in the actual number of days between enrolment and the month two visit and subsequent visits.  

**DHAD data are summarized in Table 2. Why was it not also a primary outcome?**

 Various studies including ours ^30^ have shown that children initially gain weight before height therefore we did not expect major differences in height gain in the first 60 days. When wasting and stunting co-exist in the same child, it has been observed that, whereas rapid catch-up in tissue growth commences as soon as treatment is initiated, the peak velocity for catch-up in linear growth occurs 1 to 3 months later ^31^. This delay coincides with the time taken by children to attain a weight that is appropriate for their length ^31^. The early post discharge period (within 2 months) is normally accompanied by high gains in weight compared to later time points. We also selected weight gain as primary analysis since the original trial design used severe wasting (mid-upper-arm circumference (MUAC) <11.5cm if aged ≥6 months and <11.0cm if aged 2–5 months or had oedematous malnutrition) as an enrolment criteria and not severe stunting ^32^. We have however included changes in height in the new one-year analysis.

**Table 1: In the 1 ^st^ row, it would be informative to have a summary [median (IQR)] of ages of children at 60 days (removing “at enrolment” in the row label).** This has been edited and the median [IQR] of ages of children at 60 days included.

**Table 3: The title is long and difficult to understand. Mention is made of correlation, but R ^2^ is reported. Please shorten and articulate specifics in the footnotes, including the exposure protein variables in each model (assuming these are the proteins reported in Figure 1 and b?).**

 The title has been changed to read *“Elastic Net regression model optimal alpha parameters and performance of proteins associated with change in growth deficits within 60 days”*. We have also changed R ^2^ to r. We have added a footnote that reads “ *Optimal alpha parameter and correlation coefficients for the EN model enumerating the correlation between DWAD and DMAD at two months and exposure protein variables (untargeted plasma proteome, and targeted cytokine/chemokines, leptin, sCD14, and CRP) extracted by the multivariate regularized models*.”  

**Figure 1: Please clarify the x-axis label in a and b as regression coefficient and in the legend, specify measurement unit for each. Findings from Figures 1e and 1f, presenting Inclusion Rates associated with DWAD and DMAD, are presently not mentioned in the Results.**

 The x-axis label in Figure 1a and 1b now reads regression coefficient. Legend has been adjusted to read “Untargeted liquid chromatography tandem mass spectrometry plasma proteins, and targeted cytokines/chemokines, Leptin, sCD14, and CRP associated with DWAD ( ***a*** **) and DMAD ( ***b*** **) in multivariate elastic net (EN) regularized linear regression models at two months. Log normalised protein values were used in the analysis and regression models were adjusted for age, randomisation arm, sex, respective enrolment growth deficits, and site.”

 The findings from Figure 1e and 1f are discussed under the subheading “ ***Bootstrap analysis***. *After 1000 bootstrap iterations, only IL17a was identified in >60% of the DWAD model repetitions (* *Figure 1e* *) indicating that this was the most robust feature associated with weight gain. Using similar iterations during bootstrap validation for the DMAD model, no features were extracted at the 60% threshold and the most frequently selected features were IL17a (55%), B2M (55%), AGT (49%), SAP (48%), and sCD14 (48%) as shown (* *Figure 1f* *)*.”

                

**Discussion**

**Three-quarters down the 1 ^st^ paragraph, noting the height deficit did not show evidence of recovery, could this be placing too high an expectation on long bone growth recovery commensurate with regaining soft tissue accretion within only 60 days?**

 Our group ^30^ and others have observed that during rehabilitation of severely malnourished children who were both wasted and stunted, weight gain is rapid and appetite is high until when such a child attain a weight appropriate for their length  height/length gain ^31^.

**Few typos:**

**Figure 1: Within the parentheses (d and f) should be (e and f);**

 This has now been corrected as suggested.

**In discussion – LBP is a plasma protein that binds...**

 This has now been corrected as suggested.

**References**

 1. Njunge J, Gwela A, Kibinge N, Ngari M, et al.: Biomarkers of post-discharge mortality among children with complicated severe acute malnutrition. *Scientific Reports*. 2019; **9** (1). Publisher Full Text

 2. Lee S, West K, Cole R, Schulze K, et al.: Novel Plasma Proteins in Nepalese School-aged Children are Associated with a Small Head Size at Birth. *Scientific Reports*. 2018; **8** (1). Publisher Full Text

 3. Lee SE, Stewart CP, Schulze KJ, Cole RN, et al.: The Plasma Proteome Is Associated with Anthropometric Status of Undernourished Nepalese School-Aged Children. *J Nutr*. **147** (3): 304-313 PubMed Abstract | Publisher Full Text

 1              Sauder, M. W. *et al.* Inflammation throughout pregnancy and fetal growth restriction in rural Nepal. *Epidemiology and Infection* **147**, e258, doi:10.1017/S0950268819001493 (2019).

 2              Hindle, L. J. *et al.* Effect of multiple micronutrient supplementation during pregnancy on inflammatory markers in Nepalese women. *The American journal of clinical nutrition* **84**, 1086-1092, doi:10.1093/ajcn/84.5.1086 (2006).

 3              Lee, S. E. *et al.* Novel Plasma Proteins in Nepalese School-aged Children are Associated with a Small Head Size at Birth. *Scientific reports* **8**, 6390, doi:10.1038/s41598-018-24640-4 (2018).

 4              Lee, S. E. *et al.* The Plasma Proteome Is Associated with Anthropometric Status of Undernourished Nepalese School-Aged Children. *The Journal of nutrition* **147**, 304-313, doi:10.3945/jn.116.243014 (2017).

 5              Muriuki, J. M. *et al.* Estimating the burden of iron deficiency among African children. *BMC Medicine* **18**, 31, doi:10.1186/s12916-020-1502-7 (2020).

 6              Rogawski, E. T. *et al.* Use of quantitative molecular diagnostic methods to investigate the effect of enteropathogen infections on linear growth in children in low-resource settings: longitudinal analysis of results from the MAL-ED cohort study. *The Lancet. Global health* **6**, e1319-e1328, doi:10.1016/s2214-109x(18)30351-6 (2018).

 7              Prendergast, A. J. *et al.* Stunting Is Characterized by Chronic Inflammation in Zimbabwean Infants. *PLOS ONE* **9**, e86928, doi:10.1371/journal.pone.0086928 (2014).

 8              Campbell, D. I., Elia, M. & Lunn, P. G. Growth faltering in rural Gambian infants is associated with impaired small intestinal barrier function, leading to endotoxemia and systemic inflammation. *The Journal of nutrition* **133**, 1332-1338, doi:10.1093/jn/133.5.1332 (2003).

 9              Urlacher, S. S. *et al.* Tradeoffs between immune function and childhood growth among Amazonian forager-horticulturalists. *Proceedings of the National Academy of Sciences* **115**, E3914-E3921, doi:10.1073/pnas.1717522115 (2018).

 10           Jones, K. D. J. *et al.* Mesalazine in the initial management of severely acutely malnourished children with environmental enteric dysfunction: a pilot randomized controlled trial. *BMC Medicine* **12**, 133, doi:10.1186/s12916-014-0133-2 (2014).

 11           Lee, S. E., Schulze, K. & West, K. P. Rainer Gross Award Lecture 2018: The Childhood Plasma Proteome: Discovering its Applications in Public Health Nutrition. *Food and nutrition bulletin* **40**, 144-150, doi:10.1177/0379572119847851 (2019).

 12           Yende, S. *et al.* Inflammatory markers at hospital discharge predict subsequent mortality after pneumonia and sepsis. *American journal of respiratory and critical care medicine* **177**, 1242-1247, doi:10.1164/rccm.200712-1777OC (2008).

 13           Kaynar, A. M. *et al.* Effects of intra-abdominal sepsis on atherosclerosis in mice. *Crit Care* **18**, 469, doi:10.1186/s13054-014-0469-1 (2014).

 14           Yende, S. *et al.* Long-term Host Immune Response Trajectories Among Hospitalized Patients With Sepsis. *JAMA Network Open* **2**, e198686-e198686, doi:10.1001/jamanetworkopen.2019.8686 (2019).

 15           Rahmel, T. *et al.* Long-term mortality and outcome in hospital survivors of septic shock, sepsis, and severe infections: The importance of aftercare. *PLOS ONE* **15**, e0228952, doi:10.1371/journal.pone.0228952 (2020).

 16           Póvoa, P. *et al.* C-reactive protein and albumin kinetics after antibiotic therapy in community-acquired bloodstream infection. *International Journal of Infectious Diseases* **95**, 50-58, doi: https://doi.org/10.1016/j.ijid.2020.03.063 (2020).

 17           Njunge, J. M. *et al.* Biomarkers of post-discharge mortality among children with complicated severe acute malnutrition. *Scientific reports* **9**, 5981, doi:10.1038/s41598-019-42436-y (2019).

 18           Childhood Acute Illness and Nutrition (CHAIN) Network: a protocol for a multi-site prospective cohort study to identify modifiable risk factors for mortality among acutely ill children in Africa and Asia. *BMJ open* **9**, e028454, doi:10.1136/bmjopen-2018-028454 (2019).

 19           Sandige, H., Ndekha, M. J., Briend, A., Ashorn, P. & Manary, M. J. Home-Based Treatment of Malnourished Malawian Children with Locally Produced or Imported Ready-to-Use Food. *Journal of Pediatric Gastroenterology and Nutrition* **39**, 141-146 (2004).

 20           Yebyo, H. G., Kendall, C., Nigusse, D. & Lemma, W. Outpatient therapeutic feeding program outcomes and determinants in treatment of severe acute malnutrition in tigray, northern ethiopia: a retrospective cohort study. *PLoS One* **8**, e65840, doi:10.1371/journal.pone.0065840 (2013).

 21           Kangas, S. T. *et al.* Impact of reduced dose of ready-to-use therapeutic foods in children with uncomplicated severe acute malnutrition: A randomised non-inferiority trial in Burkina Faso. *PLoS medicine* **16**, e1002887, doi:10.1371/journal.pmed.1002887 (2019).

 22           Linneman, Z. *et al.* A large-scale operational study of home-based therapy with ready-to-use therapeutic food in childhood malnutrition in Malawi. *Maternal & Child Nutrition* **3**, 206-215, doi:10.1111/j.1740-8709.2007.00095.x (2007).

 23           Aguayo, V. M. *et al.* Community management of acute malnutrition (CMAM) programme in Pakistan effectively treats children with uncomplicated severe wasting. *Matern Child Nutr* **14 Suppl 4**, e12623, doi:10.1111/mcn.12623 (2018).

 24           James, P. T. *et al.* Low-dose RUTF protocol and improved service delivery lead to good programme outcomes in the treatment of uncomplicated SAM: a programme report from Myanmar. *Matern Child Nutr* **11**, 859-869, doi:10.1111/mcn.12192 (2015).

 25           Burza, S. *et al.* Community-based management of severe acute malnutrition in India: new evidence from Bihar. *Am J Clin Nutr* **101**, 847-859, doi:10.3945/ajcn.114.093294 (2015).

 26           Das, J. K., Salam, R. A., Saeed, M., Kazmi, F. A. & Bhutta, Z. A. Effectiveness of Interventions for Managing Acute Malnutrition in Children under Five Years of Age in Low-Income and Middle-Income Countries: A Systematic Review and Meta-Analysis. *Nutrients* **12**, 116 (2020).

 27           Leroy, J. L., Ruel, M., Habicht, J.-P. & Frongillo, E. A. Linear Growth Deficit Continues to Accumulate beyond the First 1000 Days in Low- and Middle-Income Countries: Global Evidence from 51 National Surveys. *The Journal of Nutrition* **144**, 1460-1466, doi:10.3945/jn.114.191981 (2014).

 28           Leroy, J. L., Ruel, M., Habicht, J.-P. & Frongillo, E. A. Using height-for-age differences (HAD) instead of height-for-age z-scores (HAZ) for the meaningful measurement of population-level catch-up in linear growth in children less than 5 years of age. *BMC Pediatr* **15**, 145-145, doi:10.1186/s12887-015-0458-9 (2015).

 29           Group, W. M. G. R. S. WHO Child Growth Standards based on length/height, weight and age. *Acta Paediatrica (Oslo, Norway: 1992). Supplement* **450**, 76-85 (2006).

 30           Ngari, M. M. *et al.* Linear growth following complicated severe malnutrition: 1-year follow-up cohort of Kenyan children. *Archives of Disease in Childhood* **104**, 229-235, doi:10.1136/archdischild-2018-315641 (2019).

 31           Ashworth, A. & Millward, D. Catch-up growth in children. *Nutrition reviews (USA)* (1986).

 32           Berkley, J. A. *et al.* Daily co-trimoxazole prophylaxis to prevent mortality in children with complicated severe acute malnutrition: a multicentre, double-blind, randomised placebo-controlled trial. *Lancet Glob Health* **4**, e464-473, doi:10.1016/S2214-109X(16)30096-1 (2016).

# Peer review: Reviewer response for version 1

**DOI:** 10.21956/wellcomeopenres.17953.r41367

**Published:** 2020-12-04

**Authors:** Sana Syed, Lubaina Ehsan

**Affiliations:** 1 Division of Pediatric Gastroenterology, Hepatology and Nutrition, Department of Pediatrics, School of Medicine, University of Virginia, Charlottesville, VA, USA; 2 University of Virginia, Charlottesville, VA, USA

The manuscript submitted by Dr. Njunge and colleagues aims to investigate the association of systemic inflammation among early post-discharge children who are severely malnourished and have had an acute illness. Overall, this manuscript presents results that would be of interest to the community of scientists and clinicians concerned with this problem, major strengths of their work are:

- Well-written and coherent manuscript which clearly outlines the work that was done.
- Strong statistical analysis of not only the weight/MUAC gain but also the absolute deficits in these measurements.
- A discussion that outlines each significant result clearly with extensive literature being referenced.
- The assessment of potential markers for growth being associated with inflammation which can pave the way for revised standards of anthropometric measurement among children within the same cohort.

However, there are a few issues in this manuscript that prevent us from recommending that this manuscript be indexed in its current state:

**Overall**

- There is a mismatch between the introduction and the discussion. While the introduction mentions RUTF and GH/IGF1 axis, we also suggest adding the background behind the assessment of the relationship of plasma proteomic and cytokine profiles with growth. The discussion then excellently discussed the individual results for cytokines and other markers although, the background for these measurements being part of the introduction will make the foundation of why this work was done even more strong.
- The proteomics and cytokines measurements in plasma were measured using liquid chromatography tandem mass spectrometry and targeted cytokines, chemokines, leptin and soluble CD14 by Luminex and ELISA; however, cytokines are known to be variable and fluctuate in different physiological locations and environments, which is a limitation of the study. We would suggest the authors describe how they overcame this limitation.

**Methods**

- It is mentioned that the data was from a nested case control study within a clinical trial (NCT00934492) that tested the efficacy of daily co-trimoxazole prophylaxis. However, there was no comment found on the potential effect of co-trimoxazole prophylaxis as a confounder on the results for systematic inflammation. We suggest that a short explanation is added, whether in the main manuscript or supplemental material.
- The authors have used a 60 day cut-off post-discharge – was there a particular reason for selecting this and not any earlier/later time-point and any literature supporting assessment of growth at 60 days post-discharge?
- We suggested clarifying how ‘absolute deficit’ was calculated.

**Results**

- Was there a difference noted for patients that were born prematurely or underweight since the eventual weight gain may have been due to regression to the mean? Any analysis that was done after excluding these patients?

**Discussion**

- A few comments are mentioned under the ‘overall’ heading.
- Any future directions for the current work?
- We also briefly suggest adding strengths of the study, although not necessary.

Is the work clearly and accurately presented and does it cite the current literature?

Yes

If applicable, is the statistical analysis and its interpretation appropriate?

Yes

Are all the source data underlying the results available to ensure full reproducibility?

Partly

Is the study design appropriate and is the work technically sound?

Yes

Are the conclusions drawn adequately supported by the results?

Yes

Are sufficient details of methods and analysis provided to allow replication by others?

Yes

Reviewer Expertise:

Gastroenterology, Nutrition, Data Science

We confirm that we have read this submission and believe that we have an appropriate level of expertise to confirm that it is of an acceptable scientific standard, however we have significant reservations, as outlined above.
